# Supplementary material for: Soil mixing with organic matter amendment improves Albic soil physicochemical properties and crop yield in Heilongjiang province, China
Source: PLoS One. 2020 Oct 13;15(10):e0239788. doi: 10.1371/journal.pone.0239788 (PMC7553284; doi:10.1371/journal.pone.0239788)
Supplement: S1 Fig — (DOCX) [file pone.0239788.s001.docx]

**S1 Fig. Fig 3. Effects of soil mixing on the soil water holding capacity of Albic soil.**

| **Treatment** | **2015** | | | **2016** | | |
| --- | --- | --- | --- | --- | --- | --- |
|  | **Ap (%)** | **Aw (%)** | **B (%)** | **Ap (%)** | **Aw (%)** | **B (%)** |
| **CS** | 37.18 ± 0.40 b | 24.31 ± 0.74 b | 31.70 ± 0.73 a | 36.65 ± 0.78 a | 24.43 ± 0.32 c | 30.89 ± 0.28 a |
| **TSMP** | 39.72 ± 0.67 a | 29.43 ± 1.02 a | 29.13 ± 0.37 b | 38.10 ± 0.31 a | 29.02 ± 1.01 ab | 29.94 ± 0.21 ab |
| **FSMP** | 38.51 ± 0.48 ab | 29.97 ± 0.81 a | 31.06 ± 0.74 ab | 37.70 ± 0.55 a | 31.02 ± 0.27 a | 29.49 ± 0.37 b |
| **TSIMP** | 38.18 ± 0.67 ab | 26.89 ± 1.22 ab | 30.48 ± 0.39 ab | 38.00 ± 1.14 a | 28.56 ± 0.72 b | 30.32 ± 0.56 ab |

Different lowercase letters indicate significant differences between samples (*P*< 0.05). Values are means ± standard errors (n=3).
