# Supplementary material for: Soil mixing with organic matter amendment improves Albic soil physicochemical properties and crop yield in Heilongjiang province, China
Source: PLoS One. 2020 Oct 13;15(10):e0239788. doi: 10.1371/journal.pone.0239788 (PMC7553284; doi:10.1371/journal.pone.0239788)
Supplement: S2 Fig — (DOCX) [file pone.0239788.s002.docx]

**S2 Fig. Fig 4. Effects of soil mixing on the soil penetration resistance of Albic soil.**

| **2015** | | | | | **2016** | | | |
| --- | --- | --- | --- | --- | --- | --- | --- | --- |
| **Soil depth** | **CS** | **TSMP** | **FSMP** | **TSIMP** | **CS** | **TSMP** | **FSMP** | **TSIMP** |
| **0** | 0.00 | 0.00 | 0.00 | 0.00 | 0.00 | 0.00 | 0.00 | 0.00 |
| **5** | 0.25 ± 0.09 a | 0.07 ± 0.02 a | 0.04 ± 0.01 a | 0.20 ± 0.10 a | 0.07 ± 0.02 a | 0.07 ± 0.02 a | 0.07 ± 0.02 a | 0.12 ± 0.04 a |
| **10** | 0.88 ± 0.07 a | 0.27 ± 0.07 b | 0.16 ± 0.07 b | 0.32 ± 0.17 b | 0.24 ± 0.09 a | 0.32 ± 0.09 a | 0.37 ± 0.09 a | 0.56 ± 0.13 a |
| **15** | 1.12 ± 0.06 a | 0.41 ± 0.10 b | 0.21 ± 0.09 b | 0.93 ± 0.18 a | 0.70 ± 0.17 a | 0.78 ± 0.24 a | 0.66 ± 0.25 a | 0.93 ± 0.12 a |
| **20** | 1.20 ± 0.06 a | 1.12 ± 0.07 a | 1.05 ± 0.06 a | 1.17 ± 0.16 a | 1.11 ± 0.17 a | 0.95 ± 0.27 a | 1.07 ± 0.19 a | 1.12 ± 0.06 a |
| **25** | 2.03 ± 0.03 a | 1.39 ± 0.04 b | 1.26 ± 0.04 b | 1.50 ± 0.13 b | 2.01 ± 0.03 a | 1.46 ± 0.03 c | 1.36 ± 0.17 bc | 1.78 ± 0.14 ab |
| **30** | 1.87 ± 0.17 a | 1.36 ± 0.10 ab | 1.35 ± 0.06 b | 1.75 ± 0.16 a | 1.79 ± 0.03 a | 1.43 ± 0.06 b | 1.56 ± 0.04 b | 1.60 ± 0.07 b |
| **35** | 1.65 ± 0.05 a | 1.41 ± 0.10 a | 1.40 ± 0.08 a | 1.51 ± 0.10 a | 1.77 ± 0.09 a | 1.56 ± 0.09 ab | 1.51 ± 0.02 b | 1.66 ± 0.04 ab |
| **40** | 1.53 ± 0.09 a | 1.36 ± 0.12 a | 1.32 ± 0.09 a | 1.51 ± 0.01 a | 1.56 ± 0.09 a | 1.68 ± 0.03 a | 1.55 ± 0.07 a | 1.48 ± 0.08 a |
| **45** | 1.47 ± 0.12 a | 1.45 ± 0.12 a | 1.32 ± 0.05 a | 1.61 ± 0.10 a | 1.53 ± 0.09 a | 1.50 ± 0.03 a | 1.48 ± 0.08 a | 1.47 ± 0.09 a |
| **50** | 1.42 ± 0.12 a | 1.41 ± 0.11 a | 1.36 ± 0.03 a | 1.52 ± 0.04 a | 1.45 ± 0.06 a | 1.46 ± 0.03 a | 1.40 ± 0.07 a | 1.36 ± 0.08 a |
| **55** | 1.37 ± 0.12 a | 1.32 ± 0.10 a | 1.39 ± 0.05 a | 1.52 ± 0.04 a | 1.36 ± 0.08 a | 1.36 ± 0.03 a | 1.33 ± 0.06 a | 1.28 ± 0.03 a |
| **60** | 1.33 ± 0.13 a | 1.29 ± 0.07 a | 1.39 ± 0.05 a | 1.48 ± 0.04 a | 1.27 ± 0.05 a | 1.29 ± 0.04 a | 1.29 ± 0.07 a | 1.24 ± 0.02 a |

Different lowercase letters indicate significant differences between samples (*P*< 0.05). Values are means ± standard errors (n=3).
