# Supplementary material for: Soil mixing with organic matter amendment improves Albic soil physicochemical properties and crop yield in Heilongjiang province, China
Source: PLoS One. 2020 Oct 13;15(10):e0239788. doi: 10.1371/journal.pone.0239788 (PMC7553284; doi:10.1371/journal.pone.0239788)
Supplement: S3 Fig — (DOCX) [file pone.0239788.s003.docx]

**S3 Fig. Fig 5. Effects of soil mixing on the water-stable aggregate of Albic soil.**

| **2015** | | | | | **2016** | | | |
| --- | --- | --- | --- | --- | --- | --- | --- | --- |
| **0-20 cm** | **>2 mm** | **2-0.25 mm** | **0.25-0.053 mm** | **<0.053 mm** | **>2 mm** | **2-0.25 mm** | **0.25-0.053 mm** | **<0.053 mm** |
| **CS** | 3.02 ± 0.03 b | 50.76 ± 1.97 b | 33.81 ± 1.90 a | 12.41 ± 0.69 a | 1.14 ± 0.02 b | 40.07 ± 1.39 b | 37.40 ± 1.37 a | 21.39 ± 0.12 a |
| **TSMP** | 3.50 ± 0.24 ab | 56.83 ± 1.49 a | 28.27 ± 1.07 a | 11.41 ± 1.97 a | 1.40 ± 0.03 a | 49.69 ± 1.20 a | 32.36 ±1.10 b | 16.55 ±0.41 b |
| **FSMP** | 3.80 ± 0.13 a | 57.41 ± 0.98 a | 28.33 ± 1.20 a | 10.46 ± 0.36 a | 1.47 ± 0.07 a | 46.20 ± 1.83 a | 34.41 ± 1.71 ab | 17.92 ± 0.53 ab |
| **TSIMP** | 3.59 ± 0.18 a | 53.49 ± 2.16 ab | 30.85 ± 2.85 a | 12.07 ± 1.71 a | 1.21 ± 0.06 b | 44.85 ± 2.48 ab | 35.27 ± 1.47 ab | 18.67 ± 2.18 ab |
| **20-40 cm** | **>2 mm** | **2-0.25 mm** | **0.25-0.053 mm** | **<0.053 mm** | **>2 mm** | **2-0.25 mm** | **0.25-0.053 mm** | **<0.053 mm** |
| **CS** | 2.47 ± 0.06 a | 46.19 ± 3.03 b | 39.32 ± 2.22 a | 12.10 ± 0.99 a | 0.68 ± 0.05 b | 39.15 ± 1.83 b | 45.03 ± 1.71 a | 15.39 ± 0.38 a |
| **TSMP** | 2.93 ± 0.25 a | 50.36 ± 1.00 ab | 36.05 ± 0.83 ab | 10.66 ± 0.28 ab | 1.51 ± 0.15 a | 47.16 ± 0.58 a | 38.58 ± 1.55 b | 12.76 ± 1.18 b |
| **FSMP** | 2.50 ± 0.13 a | 55.49 ± 0.28 a | 32.52 ± 0.31 b | 9.48 ± 0.10 b | 1.33 ± 0.39 ab | 51.09 ± 2.06 a | 32.55 ± 2.52 c | 15.03 ± 0.35 a |
| **TSIMP** | 2.75 ± 0.10 a | 48.05 ± 1.06 b | 37.38 ± 0.37 a | 11.75 ± 0.74 a | 1.38 ± 0.02 ab | 41.47 ± 0.42 b | 41.76 ± 0.63 ab | 15.13 ± 0.16 a |

Different lowercase letters indicate significant differences between samples (*P*< 0.05). Values are means ± standard errors (n=3).
