# Supplementary material for: Soil mixing with organic matter amendment improves Albic soil physicochemical properties and crop yield in Heilongjiang province, China
Source: PLoS One. 2020 Oct 13;15(10):e0239788. doi: 10.1371/journal.pone.0239788 (PMC7553284; doi:10.1371/journal.pone.0239788)
Supplement: S4 Fig — (DOC) [file pone.0239788.s004.doc]

**S4 Fig. Fig 6. Effects of soil mixing on the MWD of Albic soi**l.

| **Treatment** | **2015** | | **2016** | |
| --- | --- | --- | --- | --- |
|  | **0-20 cm** | **20-40 cm** | **0-20 cm** | **20-40 cm** |
| **CS** | 0.73 ± 0.02 b | 0.55 ± 0.01 b | 0.68 ± 0.03 b | 0.54 ± 0.02 b |
| **TSMP** | 0.81 ± 0.01 a | 0.66 ± 0.01 a | 0.73 ± 0.02 ab | 0.65 ± 0.01 a |
| **FSMP** | 0.82 ± 0.01 a | 0.63 ± 0.02 a | 0.76 ± 0.01 a | 0.68 ± 0.03 a |
| **TSIMP** | 0.78 ± 0.03 ab | 0.61 ± 0.03 ab | 0.69 ± 0.01 b | 0.58 ± 0.00 b |

Different lowercase letters indicate significant differences between samples (*P*< 0.05). Values are means ± standard errors (n=3).
